# Estimating the Fitness Advantage Conferred by Permissive Neuraminidase Mutations in Recent Oseltamivir-Resistant A(H1N1)pdm09 Influenza Viruses

**DOI:** 10.1371/journal.ppat.1004065

**Published:** 2014-04-03

**Authors:** Jeff Butler, Kathryn A. Hooper, Stephen Petrie, Raphael Lee, Sebastian Maurer-Stroh, Lucia Reh, Teagan Guarnaccia, Chantal Baas, Lumin Xue, Sophie Vitesnik, Sook-Kwan Leang, Jodie McVernon, Anne Kelso, Ian G. Barr, James M. McCaw, Jesse D. Bloom, Aeron C. Hurt

**Affiliations:** 1 World Health Organization Collaborating Centre for Reference and Research on Influenza, North Melbourne, Australia; 2 Division of Basic Sciences, Fred Hutchinson Cancer Research Center, Seattle, Washington, United States of America; 3 Molecular and Cellular Biology Program, University of Washington, Seattle, Washington, United States of America; 4 Melbourne School of Population and Global Health, The University of Melbourne, Parkville, Australia; 5 Bioinformatics Institute (BII), Agency for Science, Technology and Research (A*STAR), Singapore; 6 National Public Health Laboratory, Communicable Diseases Division Ministry of Health, Singapore; 7 School of Biological Sciences (SBS), Nanyang Technological University (NTU), Singapore; 8 Monash University, School of Applied Sciences, Churchill, Victoria, Australia; 9 Murdoch Childrens Research Institute, The Royal Children's Hospital, Melbourne, Victoria, Australia; Virginia-Maryland Regional College of Veterinary Medicine, United States of America

## Abstract

Oseltamivir is relied upon worldwide as the drug of choice for the treatment of human influenza infection. Surveillance for oseltamivir resistance is routinely performed to ensure the ongoing efficacy of oseltamivir against circulating viruses. Since the emergence of the pandemic 2009 A(H1N1) influenza virus (A(H1N1)pdm09), the proportion of A(H1N1)pdm09 viruses that are oseltamivir resistant (OR) has generally been low. However, a cluster of OR A(H1N1)pdm09 viruses, encoding the neuraminidase (NA) H275Y oseltamivir resistance mutation, was detected in Australia in 2011 amongst community patients that had not been treated with oseltamivir. Here we combine a competitive mixtures ferret model of influenza infection with a mathematical model to assess the fitness, both within and between hosts, of recent OR A(H1N1)pdm09 viruses. In conjunction with data from *in vitro* analyses of NA expression and activity we demonstrate that contemporary A(H1N1)pdm09 viruses are now more capable of acquiring H275Y without compromising their fitness, than earlier A(H1N1)pdm09 viruses circulating in 2009. Furthermore, using reverse engineered viruses we demonstrate that a pair of permissive secondary NA mutations, V241I and N369K, confers robust fitness on recent H275Y A(H1N1)pdm09 viruses, which correlated with enhanced surface expression and enzymatic activity of the A(H1N1)pdm09 NA protein. These permissive mutations first emerged in 2010 and are now present in almost all circulating A(H1N1)pdm09 viruses. Our findings suggest that recent A(H1N1)pdm09 viruses are now more permissive to the acquisition of H275Y than earlier A(H1N1)pdm09 viruses, increasing the risk that OR A(H1N1)pdm09 will emerge and spread worldwide.

## Author Summary

Antimicrobial resistance is an increasing problem for the treatment of infectious diseases. In 2007–2008 human seasonal A(H1N1) influenza viruses rapidly acquired resistance to the most commonly used anti-influenza drug oseltamivir, via a H275Y amino acid mutation within the neuraminidase (NA) protein. In 2009 the oseltamivir sensitive A(H1N1)pdm09 virus (encoding NA 275H) emerged in the human population, rapidly replacing the oseltamivir resistant seasonal A(H1N1) virus. However, there is increasing concern that currently circulating A(H1N1)pdm09 viruses may similarly acquire oseltamivir resistance (via the NA H275Y mutation) and become widespread. Here we demonstrate that two novel amino acid changes present in virtually all recent A(H1N1)pdm09 viruses (NA V241I and N369K) enable the acquisition of the NA H275Y oseltamivir resistance mutation without compromising viral fitness. As such recent A(H1N1)pdm09 viruses are now one step closer to acquiring widespread oseltamivir resistance.

## Introduction

The influenza NA inhibitor antiviral drug oseltamivir is a key element of public health defences against influenza, and was used during the early stages of the A(H1N1)pdm09 influenza pandemic to lessen the burden of disease in infected patients [1], [2]. Resistance to oseltamivir most commonly results from mutations in the NA protein. The most common oseltamivir resistance (OR) mutation detected in A/H1N1 viruses is the NA H275Y mutation. Prior to 2007, the incidence of OR influenza viruses was generally low (<1%) [3]–[7]. *In vitro* and *in vivo* virological studies demonstrated that OR seasonal A(H1N1) viruses had attenuated viral replication kinetics in cell culture, mice and ferrets [8]–[10], and therefore were considered to pose only a minimal threat to public health [8]. However in 2008, OR (H275Y) seasonal A(H1N1) viruses emerged and spread globally within 12 months, in the absence of oseltamivir selection pressure [11]–[14], clearly demonstrating that the fitness of H275Y seasonal A(H1N1) viruses was no longer compromised by the resistance mutation. Subsequent investigations revealed the presence of several “permissive” mutations (R222Q, V234M, and possibly D354G) in the NA of 2008–2009 seasonal A(H1N1) viruses that enabled the acquisition of H275Y without compromising viral fitness [15]–[17].

In 2009, the OR seasonal A(H1N1) virus was replaced by the oseltamivir-sensitive (OS) (NA 275H) A(H1N1)pdm09 virus. Since its emergence, there has been a concern that the same NA H275Y mutation may also become fixed within circulating A(H1N1)pdm09 viruses. Since 2009, virological surveillance has reported that the proportion of OR A(H1N1)pdm09 viruses encoding the NA H275Y mutation has remained around 1% globally, and for the first two years following its emergence only limited sporadic transmissions of H275Y A(H1N1)pdm09 viruses were reported between individuals in closed or near-contact settings [18]–[22]. However, in the United States [23], United Kingdom [24] and Australia [25] during 2011 there was a notable increase in the detection of OR A(H1N1)pdm09 viruses amongst community patients who had not received oseltamivir treatment. The largest cluster of cases occurred in 2011 around the city of Newcastle, within the Hunter New England region of Australia (subsequently referred to as HNE2011), where 15% of the A(H1N1)pdm09 viruses collected between May and September 2011 were OR, including a peak frequency of 24% in July [26]. Genetic analysis revealed that these viruses were virtually identical, suggesting emergence from a single source [26]. Epidemiological investigations revealed that this OR virus had spread in the near absence of oseltamivir treatment, prompting concern that these A(H1N1)pdm09 viruses may have obtained the capability to acquire the NA H275Y mutation without compromising viral fitness, much as seasonal A(H1N1) viruses had done previously.

Experiments with early A(H1N1)pdm09 viruses (from 2009) demonstrated that introduction of the H275Y mutation decreased total NA activity, largely by decreasing NA expression levels [15]. Therefore enhancing the total NA activity of viruses containing H275Y is likely to be a key factor required for the efficient replication and transmission of OR A(H1N1)pdm09 viruses. Previously, we used a computational analysis to predict that two NA mutations present in a large number of A(H1N1)pdm09 viruses sampled during 2010–2011 (V241I and N369K) and a third NA mutation which, although absent from the majority of A(H1N1)pdm09 viruses, was present in viruses from the HNE2011 cluster (N386S), could potentially offset the deleterious effect of H275Y upon the A(H1N1)pdm09 NA [26]. Subsequent analysis of more recent A(H1N1)pdm09 NA sequences submitted to GISAID and GenBank since mid 2012 revealed that V241I and N369K are now present in virtually all (>99%) of globally circulating A(H1N1)pdm09 viruses, whereas the N386S NA mutation has not been maintained in contemporary A(H1N1)pdm09 viruses (Figure S1).

Here we combine our ferret competitive mixtures model [27] and a series of *in vitro* assays with quantitative modelling to assess the relative fitness of A(H1N1)pdm09 viruses bearing these potentially permissive mutations (PPMs) and demonstrate that two of these mutations do indeed influence the fitness of OR A(H1N1)pdm09 influenza viruses.

## Materials and Methods

### Ethics statement

All experiments involving ferrets were conducted with the approval of the CSL Limited/Pfizer Animal Ethics Committee (permit numbers 791 and 801). All procedures were conducted according to the guidelines of the National Health and Medical Research Council as described in the Australian Code of Practice for the Care and Use of Animals for Scientific Purposes [28].

### Viruses

All of the viruses described were isolated at the WHO Collaborating Centre for Reference and Research on Influenza, Melbourne, Australia, through routine virus sampling as part of the WHO Global Influenza Surveillance and Response System (GISRS). The first ferret experiment used a “natural” (non-reverse genetics derived) virus pair. One of the viruses used was obtained from the cluster of OR cases that occurred during the HNE2011 outbreak [25], [26]: A/Newcastle/17/2011 (NA 275Y) (New17 OR) (for GISAID accession numbers see Table S1). The fitness of this virus was compared against an OS virus which was obtained from the same location and time period: A/Newcastle/163/2011 (NA 275H) (New163 OS). Full genome sequence analysis revealed a high degree of amino acid similarity between this pair of viruses (New17 OR:New163 OS  =  99.5%) and no amino acid differences that have previously been associated with virulence, receptor binding, antiviral resistance or other aspects of viral function, other than the OR NA H275Y mutation. For experiments using reverse-engineered virus pairs, denoted with the prefix rg (such as rgNew17), RNA was extracted and cloned from two OR A(H1N1)pdm09 viruses: the New17 OR virus from the HNE2011 outbreak, and A/Perth/261/2009 (Perth261 OR), an OR A(H1N1)pdm09 virus isolated from a hospitalised patient undergoing oseltamivir treatment during the early period of the 2009 pandemic. The NA segment of the early A(H1N1)pdm09 virus A/California/7/2009 (Cal7 OS) was also used for an *in vitro* assessment of NA expression and enzymatic activity. All growth of viruses *in vitro* was performed in Madin-Darby canine kidney (MDCK) cells (ATCC, #CCL-34), using maintenance media [Dulbecco's modified Eagle's medium (DMEM) (Sigma-Aldrich), supplemented with 2 mM L-glutamine (SAFC Biosciences), 1 M HEPES (SAFC Biosciences), 1% nonessential amino acids (NE) (SAFC Biosciences), 0.05% sodium bicarbonate (SAFC Biosciences), 2% penicillin-streptomycin (Sigma-Aldrich) and 4 μg/ml TPCK trypsin (Sigma-Aldrich)]. Following initial isolation, viruses were subjected to two rounds of plaque purification to achieve a homogenous population, as previously described [27]. Thereafter viruses were amplified once at 35°C for 48 h, and the supernatant was frozen in aliquots and stored at −80°C for use in ferret infection experiments. The infectious titre of each stock was determined by titration on MDCK monolayers and the 50% tissue culture infectious dose (TCID_50_)/ml was calculated according to the method of Reed and Muench [29].

### RNA extraction, RT-PCR and sequencing

RNA was extracted from cell culture supernatants as previously described [30]. Each influenza genome segment was amplified by RT-PCR and subjected to DNA sequencing as described by Hurt *et al*. [27]. DNA sequences obtained were translated and the deduced amino acid sequences aligned using Clone Manager v9.11 (Scientific & Educational Software).

### Reverse-engineered viruses and site-directed mutagenesis

All eight genome segments of the New17 OR and Perth261 OR viruses were amplified by RT-PCR and incorporated into the pHW2000 reverse genetics (rg) virus rescue plasmid [31] (kindly provided by Dr. Richard Webby, St Jude Children's Research Hospital, Memphis, USA). In order to investigate the effect of the NA PPMs upon viral fitness, plasmids encoding the New17 OR and Perth261 OR NA genome segments were subjected to site-directed mutagenesis using the GeneArt Site-Directed Mutagenesis System (Life Technologies) and relevant primer pairs (sequences available upon request). Following sequencing to confirm that the correct mutation had been inserted and that no other changes had been acquired, recombinant viruses were generated by transfection of all eight plasmids into a co-culture of human embryonic kidney-293T and MDCK cells, as previously described [31]. The correct NA sequence was confirmed by DNA sequencing for all generated viruses. All amino acid positions are described relative to the first methionine of the N1 NA.

### *In vitro* analysis of NA expression and activity

The NA protein surface expression and total activity of each NA variant were determined using 293T cells transfected with plasmids encoding the NA variants. The assays were performed as described in [32], with the following modifications: the IRES-mCherry was replaced with an IRES-GFP, the HA tag was replaced with a V5 tag, and the antibody used was the anti-V5 AF647-conjugated antibody (Invitrogen 45–1098) at a 1∶200 dilution. NA activity results were compared using a two-tailed *t* test.

### *In vitro* viral replication kinetics

In order to assess the impact of each mutation upon viral replication efficiency, each virus was subjected to single-step and multi-step replication cycle experiments. Single-step replication experiments involved the infection of MDCK cells at a high multiplicity of infection (MOI) of 1 TCID_50_/cell, followed by sampling of the cell-culture supernatant every 2 h for 12 h, while multi-step replication experiments involved the infection of cells at a low MOI of 0.01 TCID_50_/cell, followed by sampling of the cell-culture supernatant every 12 h until 60 h.

Viruses were also assayed for *in vitro* replication in the presence and absence of 250 nM oseltamivir carboxylate (kindly provided by Hoffmann-La Roche, Switzerland) as described by Yen *et al.* [33]. Briefly, confluent monolayers pre-treated for 2 h with either PBS or PBS containing 250 nM oseltamivir were infected at a MOI of 0.001 TCID_50_/cell with each virus for 30 min. Immediately thereafter excess virus particles were removed [34]. Cell monolayers were washed once with 0.9% aqueous NaCl (pH 2.2) to remove unbound virus particles, followed by two washes with PBS to adjust the pH back to pH 7.2. Cells were then grown in the presence or absence of 250 nM oseltamivir in maintenance media for 48 h, after which the cell culture supernatant was sampled and frozen at −80°C prior to subsequent titration on MDCK cells.

### *In vitro* NA inhibition assay

An *in vitro* NA inhibition assay was performed as previously described by Hurt *et al.* [35], to determine the concentration of oseltamivir required to inhibit 50% of the NA activity (IC_50_) of each virus using a logistic curve fit program (Robosage, Glaxo-SmithKline, UK).

### Ferret experiments

The relative fitness of virus pairs was assessed using a ferret competitive mixtures model previously described by our laboratory [27]. In preparation for inoculation into ferrets, each pair of viruses was diluted to 1×10^5^ TCID_50_/ml and used to inoculate ferrets either as pure populations (Virus A:Virus B, 100:0%, 0%:100%) or as a series of deliberately prepared mixtures (Virus A:Virus B, 80%:20%, 50%:50%, 20%:80%) based on their infectivity titre [27]. Each of the pure population or mixtures was considered an experimental group. Two groups of ferrets were used to assess the 50%:50% mixture. Each experimental group comprised three ferrets; one ferret served as an artificially infected donor ferret and two ferrets served as sequentially naturally infected recipient ferrets (the 1^st^ recipient was infected by the donor and the 2^nd^ recipient was infected by the 1^st^ recipient). On day 0 of each experiment, donor ferrets were anesthetised intramuscularly with 20 mg/ml Ilium Xylazil-20 (Troy-Laboratories, Australia) and intranasally inoculated with 0.5 ml PBS containing 5×10^4^ TCID_50_ of virus. Once inoculated, each donor ferret was housed separately in a high efficiency particulate air (HEPA) filtered cage. After 24 h, 1st recipient ferrets were co-housed with the donor ferrets to allow virus transmission. Beginning on day 1 post-infection (pi) of the donor ferret, all ferrets were nasal washed daily, as described [27]. Nasal washes collected from 1st recipient ferrets were analysed immediately following collection for influenza A virus using a rapid point-of-care test (Directigen EZ Flu A+B, Becton Dickinson and Company). Once influenza infection was confirmed in a 1st recipient ferret it was transferred to a clean cage housing a 2nd recipient ferret to allow virus transmission to the 2nd recipient ferret. All ferrets were euthanized on days 10 or 11 pi. All ferrets used in these experiments were 6–12 months old, of mixed gender, and all received food and water *ad libitum*. Prior to infection all ferrets were confirmed as seronegative to currently circulating human influenza viruses using a haemagglutination inhibition assay.

### Titration of infectious virus on MDCK cells

To determine the infectious virus titre, samples were titrated on MDCK cells and incubated at 37°C in 5% (v/v) CO_2_ as described by Hurt *et al*. [27] with the modification that, after 4 days of incubation the presence of haemagglutinating virus in each well was assessed using turkey red blood cells, and the virus titre calculated according to the method of Reed and Muench [29].

### Quantitative analysis of viral RNA in ferret nasal washes

A TaqMan one-step quantitative RT-PCR assay capable of detecting the influenza matrix gene segment [36] was used to detect influenza viral RNA in ferret nasal washes. This assay was performed using a SensiFast Probe Lo-ROX One-Step kit (Bioline) and an Applied Biosystems 7500 Fast Real Time PCR System (Life Technologies). Cycle threshold (Ct) values for each sample were compared to those obtained for a set of RNA transcripts encoding the A/California/7/2009 A(H1N1)pdm09 matrix genome segment, which were included as a control in each assay. These RNA transcripts were generated using the pGEM-A/Cal/7/2009 matrix plasmid kindly provided by Heidi Peck (WHO Collaborating Centre for Reference and Research on Influenza, Melbourne, Australia).

### Molecular analysis of virus mixture proportions

The relative proportion of each virus in ferret nasal washes was determined using pyrosequencing allele quantitation analysis. Viral RNA was extracted from nasal washes as described above and subjected to RT-PCR using a MyTaq One-Step RT-PCR kit (Bioline) and the primer pairs shown in Table S2. Each RT-PCR assay produced double-stranded DNA fragments which encompassed the NA H275Y, V241I or N369K mutation sites. Single-stranded biotinylated DNA was purified from each RT-PCR product and subjected to pyrosequencing analysis using the relevant sequencing primer (Table S2) and a PyroMark ID pyrosequencer (Biotage) as described by Deng *et al.* [36]. In Deng *et al.* [36], we showed that the difference between the pyrosequencing estimate and the known proportion of H275 vs. Y275 in pure viruses and defined mixtures was between 0.5 and 10.5%. Following validation of the assays used in this study we determined a similar accuracy range using purified plasmids (at a concentration range of 10^−2^ to 10^−6^ DNA copies) for each of the NA H275Y, V241I or N369K pyrosequencing assays. Therefore assay of a pure viral population may indicate the presence of a minor (<10%) proportion of the alternative viral population.

### Quantitative assessment of virus fitness differences using a mathematical model of viral replication

Within the competitive-mixtures model, fitness differences between competing strains arise in two contexts: within-host replication kinetics and host-to-host transmission. The *overall* fitness of one strain compared to another arises as a combination of these two factors. Here we developed a within-host mathematical model of virus kinetics to provide a quantitative estimate of the relative within-host viral replication fitness of the strains used in the competitive mixtures experiments. We assessed the transmission fitness of strains using our previously developed model [27], [37].

Briefly, we modelled the within-host replication of the two influenza strains using the classic Target cell – Infectious cell – Virus (TIV) paradigm [38]–[40]. Free infectious virus infects healthy ‘target’ (epithelial) cells, which following a latent phase, become infectious, releasing progeny virus particles that subsequently infect further target cells. Free virus is removed from the system due to natural decay and (time-independent) immune responses. Our particular model extends the standard TIV paradigm through inclusion of two co-infecting strains that compete for the same target cell reservoir (see also [41]). Furthermore, by modelling both infectious and total (infectious and non-infectious) viral matter [42], our model may be fitted to both TCID_50_ and RNA based assay data, providing more precise estimates of relevant within-host parameters. The experiments investigated mutations within the NA gene, and a primary function of NA is to aid in the release of budded viruses from the surface of infected cells [43], [44]. We therefore assumed that observed differences in within-host viral kinetics between strains arose through a difference in the production rate of infectious virus from infected cells. The ratio of this estimated production rate by strain served as a measure of the *relative* within-host replication fitness of the two strains. Further details of the mathematical model and how it was fitted to the available data are presented in Supplementary Text S1.

Using the within-host and transmission models we calculated the relative within-host and relative transmission fitness values along with 95% confidence intervals (95% CI) for the virus pair used in each ferret experiment. Relative fitness values of >1 indicated an advantage for virus B over A in each comparison pair, provided that the 95% CI did not cross 1.

### Database analysis of the potentially permissive mutations

All available A(H1N1)pdm09 NA protein sequences derived from viruses that infected human hosts were downloaded from the Global Initiative on Sharing All Influenza Data website (http://www.gisaid.org) (Supplementary Text S2) and the influenza virus resource at the National Centre for Biotechnology Information [45]. After removal of duplicate sequences for unique viral strains, 14234 sequences were aligned using MAFFT [http://www.ncbi.nlm.nih.gov/pubmed/18372315]. To generate the figure that illustrates the evolution timeline of the NA V241I and N369K mutations, the NA sequence from A/California/07/2009 was used as reference and the percentages of occurrences for each of the mutations were calculated on a monthly basis since April 2009. To keep the figure legible, only mutations found in 100% of all circulating viruses in any of the months since April 2009 were retained. The drug resistance H275Y and the N386S mutations were also kept for reference.

## Results

### Confirmation of oseltamivir susceptibility/resistance

An in vitro NA inhibition assay was used to assess the oseltamivir susceptibility of the HNE2011 OR and OS viruses. As expected, New17 OR (which encoded NA 275Y) had an approximately 115-fold higher IC50 compared to New163 OS (Table S3). Cell culture experiments further confirmed that oseltamivir had no effect on the replication of the New17 OR virus whereas it impaired the replication of the New163 OS virus (Figure S2).

### *In vivo* fitness comparison of ‘natural’ OR and OS HNE2011 A(H1N1)pdm09 viruses

**Figure 1:**
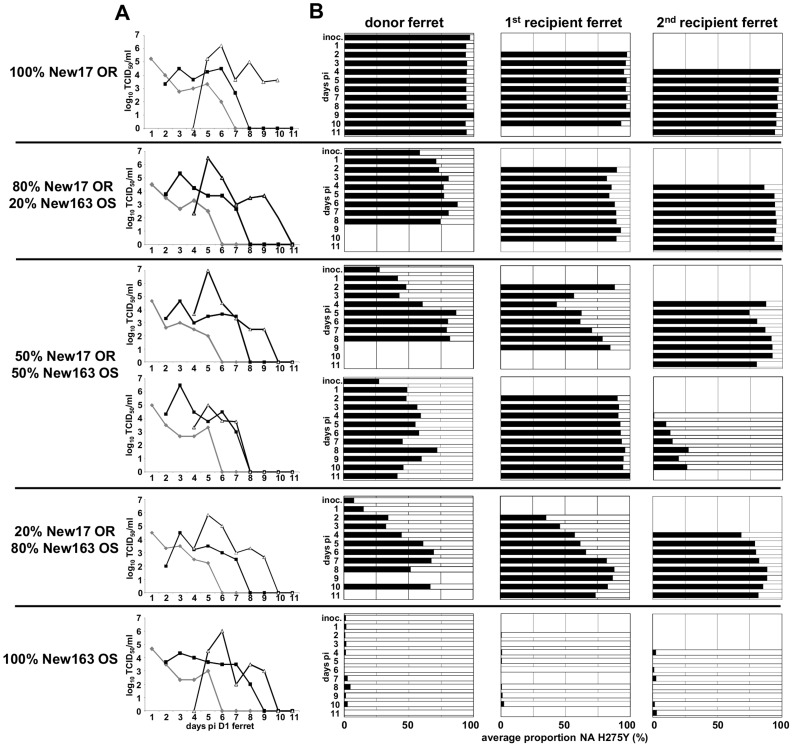
The H275Y mutation does not compromise the fitness of HNE2011 A(H1N1)pdm09 viruses. Donor ferrets were infected with pure populations or virus mixtures of A/Newcastle/17/2011 oseltamivir resistant (New17 OR) and A/Newcastle/163/2011 oseltamivir sensitive (New163 OS). Daily nasal washes from donor and 1^st^ and 2^nd^ recipient ferrets were assayed to measure the viral replication and transmission kinetics of each virus mixture/pure population and to assess the relative proportions of each virus within mixtures. (A) The infectious virus titre in each nasal wash was determined by titration on MDCK cells. (B) The relative proportions of New17 OR virus encoding NA 275Y (black bars) and New163 OS virus encoding NA 275H (white bars) in each nasal wash were determined by pyrosequencing. (A) Virus in donor ferrets (grey), 1st recipient ferrets (black lines solid squares), 2nd recipient ferrets (black lines, white triangles).

Given the spread of HNE2011 H275Y OR viruses, we hypothesised that they may have had equivalent or superior fitness to similar OS strains. To test this hypothesis we used the ferret competitive mixtures model to assess the relative fitness of an HNE2011 OR virus (New17 OR) compared with an OS virus from the same location and time (New163 OS). Following viral inoculation of ferrets, pure populations of the New17 OR and New163 OS viruses replicated to equivalent titres (in groups of three ferrets, mean [±SD] peak titres for the two viruses were 5.3±0.9 and 5.0±0.9 log_10_TCID_50_/ml respectively) with a mean [±SD] duration of shedding of 6.0±0.0 vs. 5.7±1.2 days respectively (Figure 1A, S3). Furthermore there were no significant differences in mean weight loss, mean temperature increases or other clinical signs amongst the groups of ferrets inoculated with pure populations (data not shown). Pyrosequencing analysis showed the maintenance of pure populations (*i.e.* values were >90% and within the expected variability of the assay – see materials and methods) (Figure 1B). In the four groups of ferrets inoculated with virus mixtures, a pure population of OR virus (>90%) was observed by the end of the infection in the 2^nd^ recipient of the 80∶20 group and the 1^st^ recipient of one of the 50∶50 groups (Figure 1B). Mixtures persisted until the end of the infection in the other ferrets. Mathematical analysis of the data found that, while there was no significant difference in transmissibility, the OR virus had enhanced within-host viral replication fitness (relative within-host fitness value [95% CI] = 1.07 [1.02; 2.59]) relative to the OS virus (Table 1). The transmission event between the 1^st^ and 2^nd^ recipient of one of the 50∶50 groups showed an unusual transfer from >90% OR to <5% OR virus. Sequence analysis did not reveal any new mutations within the NA genes of this virus pair, although a HA P154S amino acid change was detected in the virus mixture within the nasal washes of the 2^nd^ recipient ferret in this group. This change was not present in any of the nasal washes from the donor or 1st recipient ferrets.

**Table 1:** Relative within-host and transmission fitness of virus pairs used in ferret experiments.

			Within-host fitness		Transmission fitness	
Natural/rg viruses	Virus A	Virus B	Relative fitness value (95% CI)^c^	Virus with greater fitness	Relative fitness value (95% CI)^d^	Virus with greater fitness
Natural	New163 OS^a^	New17 OR^b^	1.07 (1.02; 2.59)	B	3.78 (0.83; 17.12)	Equivalent fitness^e^
rg	rgNew17 I241V, K369N OR	rgNew17 OR	1.82 (1.35; 7.46)	B	2.69 (1.00; 7.24)	B
rg	rgNew17 I241V OR	rgNew17 OR	3.96 (1.17; 6.83)	B	2.22 (1.24; 3.97)	B
rg	rgNew17 K369N OR	rgNew17 OR	3.14 (1.17; 5.30)	B	2.56 (0.93; 7.10)	Equivalent fitness
rg	rgPerth261 OR	rgPerth261 V241I, N369K OR	1.86 (1.37; 7.24)	B	1.13 (0.80; 1.59)	Equivalent fitness

a, OS  =  Oseltamivir sensitive.

b, OR  =  Oseltamivir resistant due to the NA H275Y mutation.

c, Relative within-host fitness: values >1 indicate advantage for virus B.

d, Relative transmission fitness: Values >1 indicate advantage for virus B.

e, No significant difference is observed when the confidence intervals cross 1.

### V241I and N369K PPMs enhance NA activity *in vitro*

**Figure 2:**
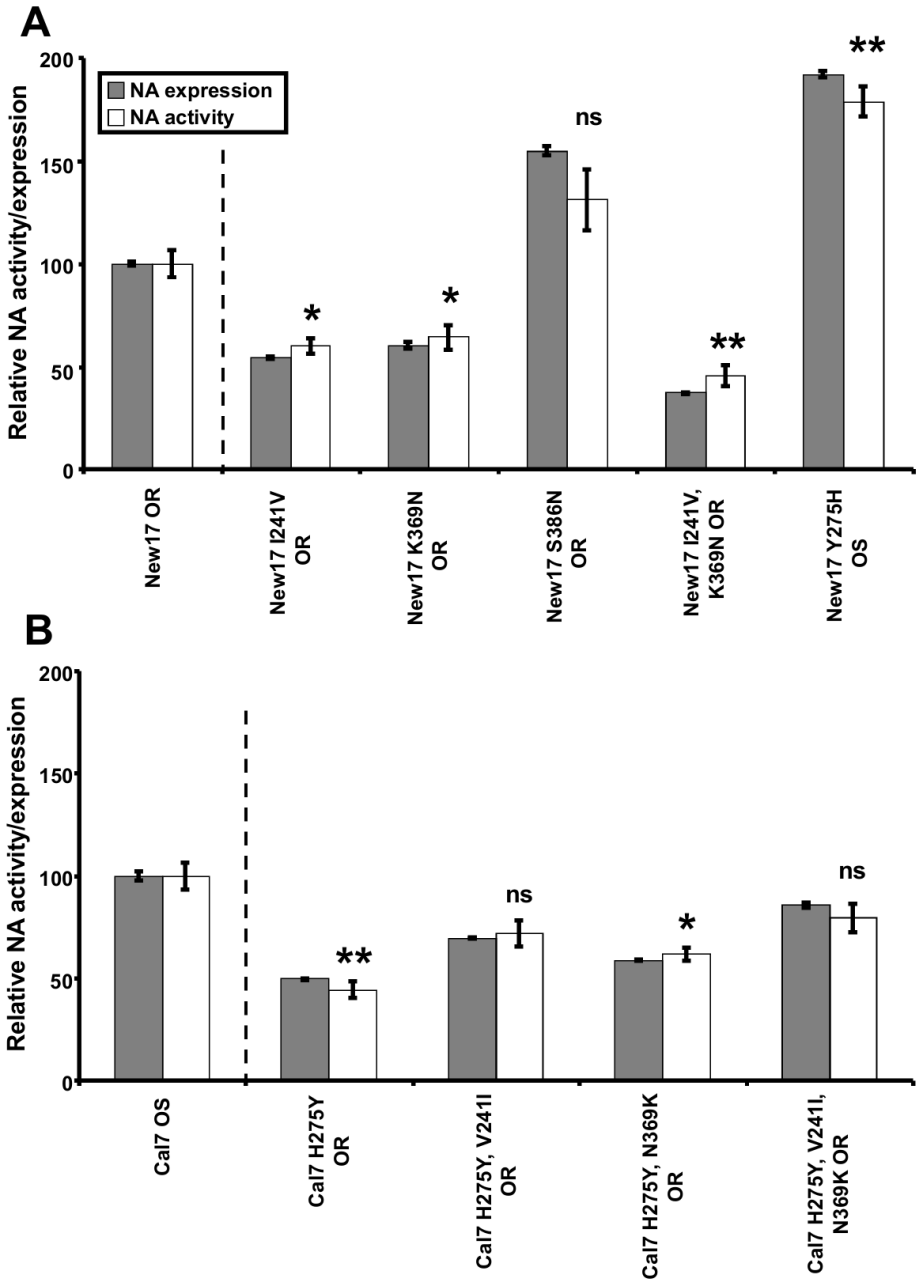
NA V241I and N369K enhance the expression and activity of A(H1N1)pdm09 NA proteins. Surface expressed NA protein and enzymatic activity was assessed 20-transfection of 293T cells with New17 OR (A), or A/California/7/2009 oseltamivir sensitive (Cal7 OS) (B), NA expression plasmids containing a C-terminal V5 epitope tag followed by an IRES-GFP, and encoding the amino acid mutations indicated. NA expression was assessed by flow cytometry after staining with a fluorescently conjugated anti-V5 antibody and gating on the GFP positive (transfected) cells. Enzyme activity was assessed by the *in vitro* NA inhibition assay. All results represent the mean and standard error of three replicate transfections and are normalised to the mean fluorescent intensity/NA activity of cells transfected with the New17 OR (A) or Cal7 OS (B) wild type NA plasmid. NA activity results for each group were compared to the relative NA activity of New17 OR, for groups shown in A, and Cal7 OS for groups shown in B, using a two-tailed *t* test. *  = *P*≤0.05, ** = *P*≤0.01, ns = *P*>0.05.

An *in vitro* NA expression system was used to investigate the effect of the NA mutations V241I, N369K and S386N on NA surface expression and enzymatic activity. Reversal of the V241I and N369K PPMs individually from the New17 OR NA protein (New17 I241V OR and New17 K369N OR) resulted in approximately 40% and 35% reductions in NA surface expression and enzymatic activity respectively (Figure 2A). However, reversal of both mutations reduced NA expression and activity by approximately 60% (Figure 2A). In contrast, removal of the N386S mutation from the HNE2011 NA (New17 S386N OR) enhanced the activity and surface expression by approximately 50% (Figure 2A), demonstrating that this mutation was unlikely to be having a beneficial effect upon the fitness of the New17 OR virus. Hence, subsequent investigations were restricted to the NA V241I and N369K mutations. Removal of H275Y from the New17 OR NA protein enhanced its surface expression and enzymatic activity by approximately 50% (Figure 2A), demonstrating the detrimental effect of the H275Y mutation upon surface expression and enzymatic activity.

To further evaluate the impact of both H275Y and the PPMs V241I and N369K *in vitro*, we introduced the mutations into an early A(H1N1)pdm09 OS virus from 2009. While the incorporation of H275Y into the Cal7 OS NA protein reduced its surface expression and activity by 50%, addition of the V241I and N369K mutations partially offset these losses, by approximately 40% and 20% respectively (Figure 2B). Addition of both V241I and N369K together offset these losses by approximately 70% (Figure 2B).

### Dual V241I and N369K PPMs enhance the *in vitro* activity and fitness of contemporary A(H1N1)pdm09 viruses in ferrets

We then determined the effect of removing both V241I and N369K from a HNE2011 OR virus on *in vivo* within-host and transmission fitness. Initial comparison of *in vitro* replication kinetics at low and high MOI showed that replication of the rgNew17 I241V, K369N OR virus was delayed for the first 4-6 h pi compared to the rgNew17 OR virus (Figure S4A, B).

**Figure 3:**
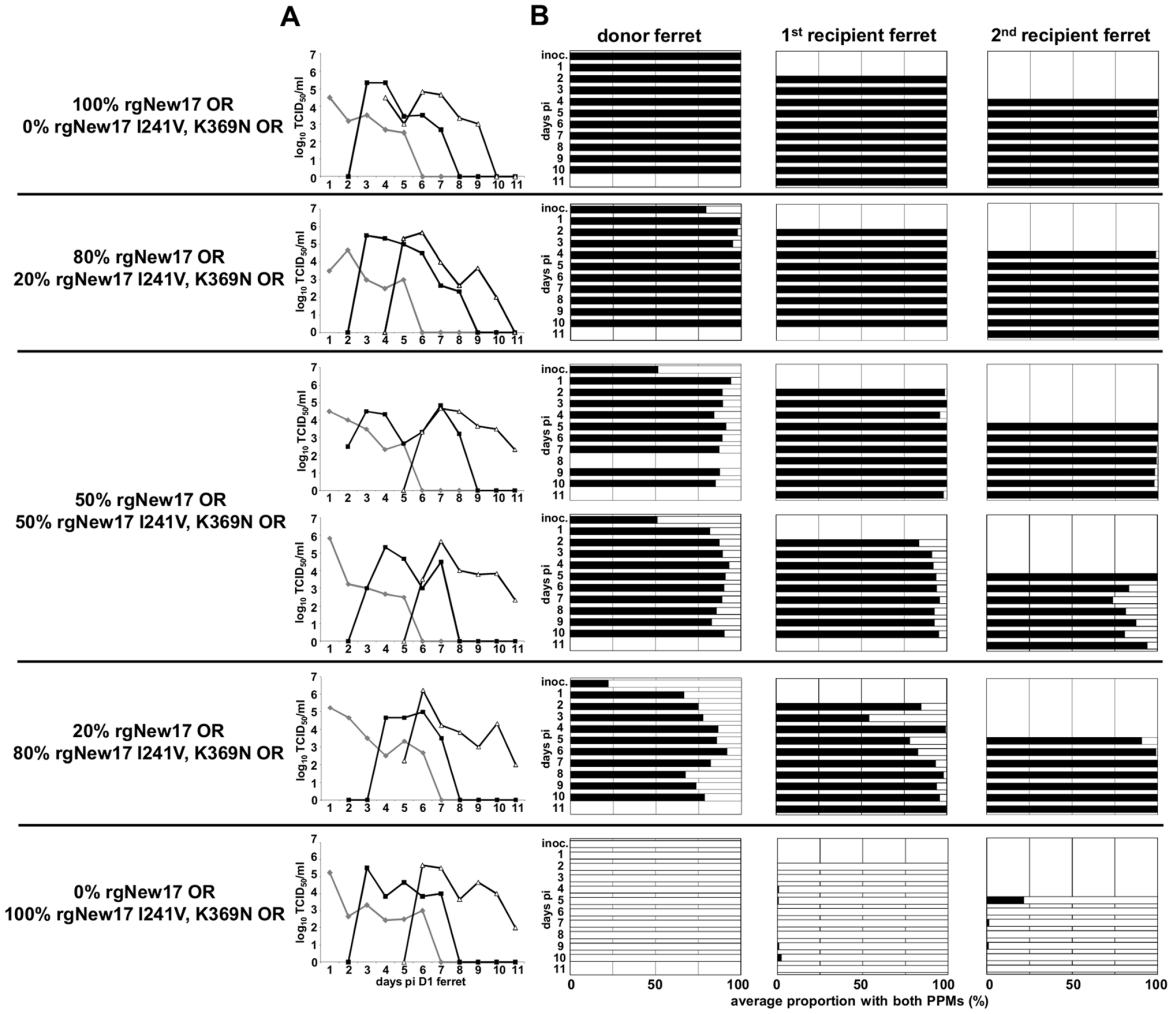
Removal of NA V241I and N369K decreases the fitness of recent H275Y A(H1N1)pdm09 viruses. Donor ferrets were infected with pure populations or virus mixtures of reverse genetics derived New17 OR (rgNew17 OR) and rgNew17 I241V, K369N OR. Daily nasal washes from donor, and naive 1st and 2nd recipient ferrets were assayed to measure the viral replication and transmission kinetics of each virus mixture/pure population and to assess the relative proportions of each virus in mixtures. (A) The infectious virus titre in each nasal wash was determined by titration on MDCK cells. (B) The relative proportions of virus encoding NA 241I, 369K (black bars) and NA 241V, 369N (white bars) in each nasal wash were determined by pyrosequencing. (A) Virus in donor ferrets (grey), 1st recipient ferrets (black lines solid squares), 2nd recipient ferrets (black lines, white triangles).

Following inoculation in ferrets, there were no significant differences in morbidity between the groups of ferrets inoculated with pure populations of the rgNew17 OR and rgNew17 I241V, K369N OR viruses (data not shown), and both viruses replicated to similar titres (4.9±0.4 and 5.5±0.3 log_10_TCID_50_/ml respectively) and were shed for an equivalent duration (5.3±0.6 days for both viruses) (Figure 3A, S5). Pyrosequencing analysis revealed that a pure virus population was maintained in each of these groups (Figure 3B). In the four groups of ferrets inoculated with virus mixtures, a pure population of rgNew17 OR virus, that was maintained upon subsequent transmission to further recipient ferrets, was observed by the end of the infection in the 1^st^ recipient of both of the 50∶50 groups and the 20∶80 group, as well as in the donor of the 80∶20 group (Figure 3B). Modelling indicated that the rgNew17 OR virus exhibited significantly superior transmission (relative fitness value [95% CI] = 2.69 [1.00; 7.24]) and within-host viral replication fitness (relative fitness value [95% CI] = 1.82 [1.35; 7.46]), compared to the rgNew17 I241V, K369N OR virus (Table 1), demonstrating that removal of NA 241I and 369K impaired the ability of the rgNew17 I241V, K369N OR virus to out-compete the rgNew17 OR virus both during replication within hosts and upon transmission between them.

### Effects of individual V241I or N369K PPMs on in vitro activity and viral fitness in ferrets

To investigate the individual influence of the NA V241I and N369K mutations upon the fitness of recent OR A(H1N1)pdm09 viruses, rgNew17 OR was compared with isogenic rg viruses from which either the NA V241I (rgNew17 I241V OR) or N369K (rgNew17 K369N OR) mutations had been removed. The *in vitro* replication kinetics of rgNew17 OR, rgNew17 I241V OR and rgNew17 K369N OR viruses along with the “natural” New17 OR virus was first determined in MDCK cells at a low and high MOI. All viruses replicated efficiently in MDCK cells with most of the New17 OR recombinant viruses following a similar growth pattern to that of the “natural” New17 OR virus from which they were derived (Figure S4A, B). However, rgNew17 K369N OR showed delayed replication for the first 6–8 h pi at a high MOI (Figure S4A), suggesting that removal of NA 369K had a somewhat detrimental effect upon virus growth *in vitro*.

Analysis of the rgNew17 OR vs. rgNew17 I241V OR virus pair in ferrets showed that pure populations of the two viruses replicated to 4.0±0.5, and 5.2±0.4 log_10_TCID_50_/ml respectively, and were shed for 5.3±1.2 and 6.0±1.0 days respectively (Figure S6A, C). In addition, there were no significant differences in morbidity between the groups of ferrets inoculated with the pure populations (data not shown). Pyrosequencing analysis revealed that a pure virus population had been maintained in each of these groups (Figure S6B). In the four groups of ferrets inoculated with virus mixtures, a pure population of rgNew17 OR virus, that was maintained upon subsequent transmission to further recipient ferrets, was observed by the end of the infection in the 1^st^ recipient of one of the 50∶50 groups and the 80∶20 group. Modelling revealed that the rgNew17 OR virus exhibited significantly superior transmission (relative transmission fitness value [95% CI] = 2.22 [1.24; 3.97]) and within-host viral replication fitness (relative within-host fitness value [95% CI] = 3.96 [1.17; 6.83]) compared to the rgNew17 I241V OR virus (Table 1), demonstrating that the V241I NA mutation is important in contemporary A(H1N1)pdm09 OR viruses for both within-host and transmission fitness.

To assess the effect of the K369N mutation, ferrets were inoculated with the rgNew17 OR and rgNew17 K369N OR viruses. Pure populations of the rgNew17 OR and rgNew17 K369N OR viruses replicated to 5.0±0.5 and 4.1±0.6 log_10_TCID_50_/ml respectively, and both viruses were shed for a similar duration of 5.0±1.2 and 5.3±0.6 days respectively (Figure S7A, C). Furthermore, there were no significant differences in morbidity between the groups of ferrets inoculated with the pure populations (data not shown). Pyrosequencing analysis revealed that a pure virus population had been maintained in each of these groups (Figure S7B). In the four groups of ferrets inoculated with virus mixtures, a pure population of rgNew17 OR virus was observed by the end of the infection in the 1^st^ recipient of both of the 50∶50 groups and the 80∶20 group, as well as in the 2^nd^ recipient of the 20∶80 group. Virus transmitted to subsequent recipient ferrets in these groups persisted as a pure population of rgNew17 OR virus, with the exception of a 2^nd^ recipient ferret in one of the 50∶50 groups in which rgNew17 OR virus accounted for 87% of the total virus present in the nasal wash on the final day of the experiment. Modelling found that, while the rgNew17 OR virus exhibited significantly superior within-host viral replication fitness compared to the rgNew17 K369N OR virus (relative within-host fitness value [95% CI] = 3.14 [1.17; 5.30]), there was no statistical evidence for a transmission fitness difference between the viruses (Table 1).

### V241I and N369K PPMs enhance in vitro activity and viral fitness of early A(H1N1)pdm09 viruses in ferrets

**Figure 4:**
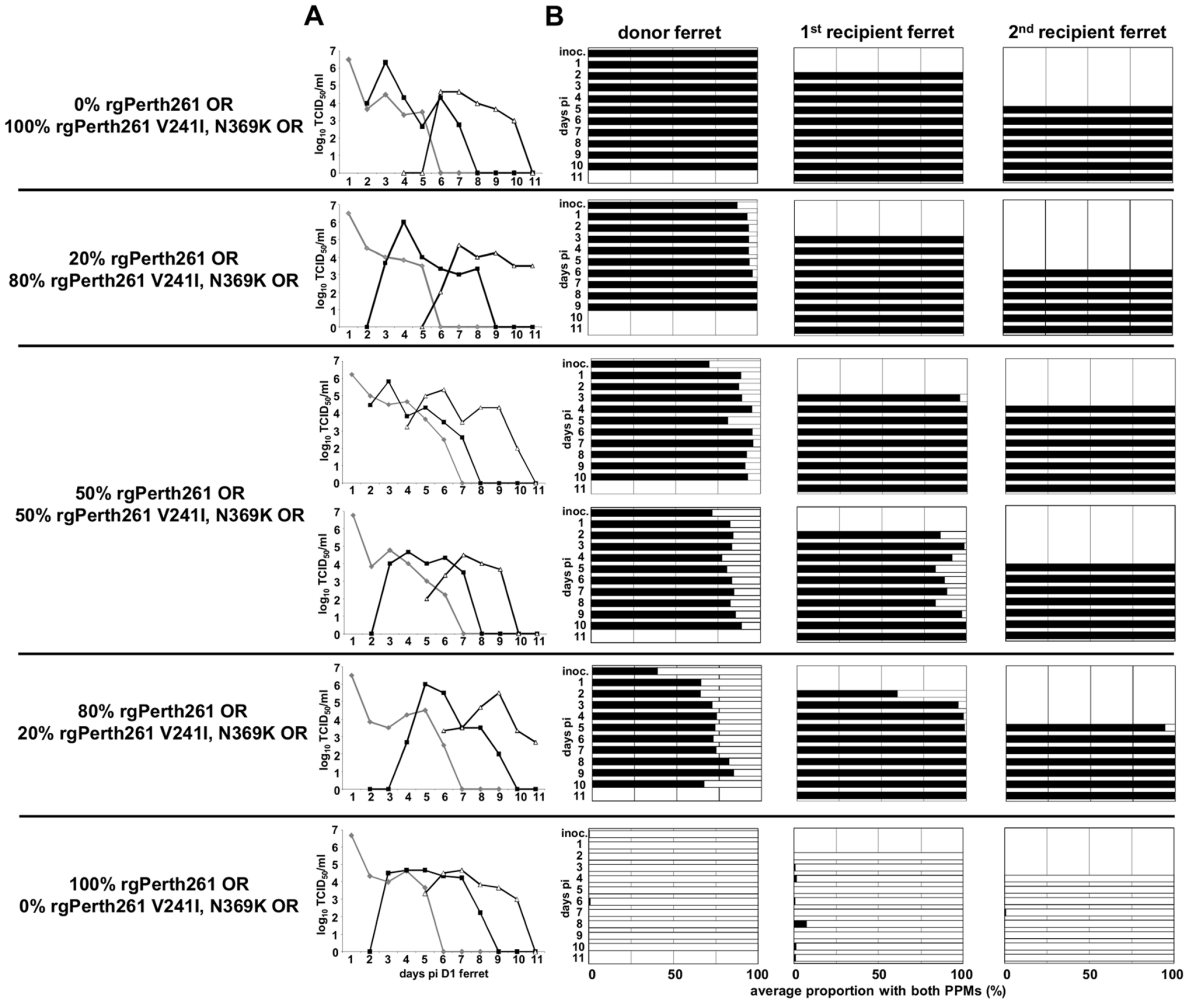
Addition of NA V241I and N369K enhances the fitness of earlier H275Y A(H1N1)pdm09 viruses. Donor ferrets were infected with pure populations or virus mixtures of reverse genetics derived A/Perth/261/2009 oseltamivir resistant (rgPerth261 OR) and rgPerth261 V241I, N369K OR. Daily nasal washes from donor and 1st and 2nd recipient ferrets were assayed to measure the viral replication and transmission kinetics of each virus mixture/pure population and to assess the relative proportions of each virus within mixtures. (A) The infectious virus titre in each nasal wash was determined by titration on MDCK cells. (B) The relative proportions of virus encoding NA 241I, 369K (black bars) and NA 241V, 369N (white bars) in each nasal wash were determined by pyrosequencing. (A) Virus in donor ferrets (grey), 1st recipients (black lines solid squares), 2nd recipients (black lines, white triangles).

*In vitro* assays demonstrated that introduction of the NA V241I and N369K mutations into an earlier A(H1N1)pdm09 OR NA protein, that did not naturally contain these mutations, resulted in enhanced enzymatic activity and NA expression (Figure 2). Therefore the effect of introducing these PPMs into an early A(H1N1)pdm09 OR virus (Perth261 OR) was assessed in ferrets. Rg viruses were generated encoding the complete Perth261 OR genome without any changes (rgPerth261 OR) and with the NA V241I and N369K mutations (rgPerth261 V241I, N369K OR). *In vitro* replication kinetics showed that both rg viruses grew more rapidly and to higher virus titres than the “natural” parent (Perth261 OR) virus, at both a low and high MOI (Figure S4C, D). Following inoculation into ferrets, there were no significant differences in morbidity between the groups of ferrets inoculated with pure populations of rgPerth261 OR and rgPerth261 V241I, N369K OR (data not shown), and both viruses replicated to similar titres of 5.8±1.0 and 5.4±1.2 log_10_TCID_50_/ml respectively, and were shed for a similar duration of 5.3±0.6 and 5.7±0.6 days respectively (Figure 4A, S8). Pyrosequencing analysis revealed that a pure virus population was maintained in each of these groups (Figure 4B). In the four groups of ferrets inoculated with virus mixtures, a pure population of rgPerth261 V241I, N369K OR virus, that was maintained upon subsequent transmission to further recipient ferrets, was observed by the end of the infection in the donor in the 20∶80 group, and in the 1^st^ recipient in the 80∶20 group, and both of the 50∶50 groups (Figure 4B). Modelling showed that while the rgPerth261 V241I, N369K OR virus exhibited superior within-host viral replication fitness (relative within-host fitness value [95% CI] = 1.86 [1.37; 7.24]) compared to the rgPerth261 OR virus, there was no significant difference in transmission fitness between the viruses (Table 1). Hence in the case of this experiment, the lack of any rgPerth261 OR virus in the 2nd recipient ferrets in each of the groups inoculated with virus mixtures, demonstrates how a virus with superior within-host replication fitness but with equivalent transmission fitness, may replicate more efficiently within successive infected hosts, such that its relative proportion increases prior to each transmission event, eventually dominating the virus mixture.

## Discussion

The PPMs NA V241I and N369K are now present in >99% of circulating A(H1N1)pdm09 viruses (Figure S1). The experiments and accompanying mathematical analysis presented in this study demonstrate that these mutations enable A(H1N1)pdm09 viruses to maintain robust viral fitness when they acquire the NA H275Y oseltamivir resistance mutation. The computational analysis reported previously [26], in conjunction with the *in vitro* experiments presented here, demonstrate that NA V241I and N369K are indeed permissive mutations that act by enhancing both the surface expression and total activity of H275Y A(H1N1)pdm09 NA proteins, similar to the effect that the R222Q and V234M mutations had on the NA of H275Y seasonal A(H1N1) viruses [15].

The data obtained from the competitive-mixture ferret experiments performed in this study were subjected to mathematical modelling to determine the relative within-host and transmission fitness of each virus pair. In this way it was possible to investigate whether the presence or absence of the NA V241I and/or the N369K permissive mutations enabled the OR viruses to replicate more efficiently within ferrets, and/or be more efficiently transmitted between ferrets.

In using the within-host mathematical model to calculate the relative within-host viral replication fitness (summarised in Table 1) we made the assumption that the observed strain-dependence in viral kinetics arose due to differing infectious virus production rates between strains (see Supplementary Text S1 for mathematical details). While there are other plausible biological explanations for the observed within-host viral kinetics, a careful consideration of these alternatives (see Supplementary Text S1 for details) suggests they are not reconcilable with the picture of outgrowth that we see across multiple host-to-host transmission events in many of the competitive-mixtures experiments performed in this work.

Previous studies in ferrets, guinea pigs and mice revealed broadly equivalent [46]–[50], or lower fitness [51] of early H275Y OR A(H1N1)pdm09 viruses compared to genetically similar OS (NA 275H) strains. In most of these studies, the viruses used did not contain the PPMs V241I and N369K which were shown here to improve the fitness of the OR viruses. However a recent study by Abed *et al*. [50] showed that the introduction of the V241I and N369K PPMs into an early H275Y A(H1N1)pdm09 virus resulted in higher virus titres in ferret nasal washes. Abed *et al*. [50] also noted that introduction of a T289M NA mutation (which was identified as a PPM by computational analyses, but has not yet been detected in circulating strains) into an early H275Y A(H1N1)pdm09 virus resulted in greater weight loss, enhanced mortality and higher lung viral titres in mice.

Most recently in 2013 two other NA mutations (N44S and N200S) have become almost universally observed in A(H1N1)pdm09 viruses, whilst at the same time an NA V106I mutation, which was rapidly acquired at the beginning of the A(H1N1)pdm09 pandemic, has been lost (Figure S1). Whether these more recently acquired NA changes have an impact upon the fitness of OR A(H1N1)pdm09 viruses remains to be investigated.

Given the apparent robust fitness of the H275Y HNE2011 viruses in this study, the obvious question is why have they not yet re-emerged? One explanation is that a high level of circulating A(H1N1)pdm09 viruses may be required for a A(H1N1)pdm09 OR virus to become established and spread. The Australian HNE2011 virus cluster emerged [25], [26] during a season when A(H1N1)pdm09 viruses accounted for almost 40% of all influenza A and B viruses detected globally but, in 2012 and 2013, the proportion of A(H1N1)pdm09 viruses circulating has been considerably lower (9% and 25% respectively) [52]. In the most recent 2013/14 Northern Hemisphere influenza season, a cluster of A(H1N1)pdm09 H275Y OR viruses that contained both the V241I and N369K PPMs plus an additional N386K NA mutation, was detected in Sapporo, Japan [53], during a period of the season where A(H1N1)pdm09 viruses contributed approximately 50% of the circulating influenza strains [54]. Although the majority of Japanese A(H1N1)pdm09 OR viruses are currently localised to the Sapporo prefecture (as of Feb 3,2014, the frequency of resistance in Sapporo was 88% [15/17] compared with 7% [22/298] for the whole of Japan [55]), there have been reports of genetically similar A(H1N1)pdm09 OR viruses also being detected in China during the same time period [56]. Such clusters need to be closely monitored to determine if spread of OR viruses is occurring into other regions. Apart from NA PPMs, it may be that other properties, such as antigenic novelty, are also necessary for an OR virus to spread widely. In 2007–2008, the H275Y NA mutation became fixed in a new seasonal A(H1N1) antigenic variant (A/Brisbane/59/2007-like), suggesting that the antigenic novelty of the OR virus assisted its prolific spread [57]. The results of this study show that A(H1N1)pdm09 viruses have now acquired permissive NA mutations which allow them to retain viral fitness when the H275Y NA mutation is present, raising the possibility of rapid global spread of an OR A(H1N1)pdm09 virus if H275Y were to arise in an antigenically drifted virus. A(H1N1)pdm09 viruses have now been circulating in humans for over four years, but are yet to undergo a significant antigenic change (as evidenced by the continued inclusion of A/California/7/2009 in the human seasonal influenza vaccine since 2009). As the H1 component of the vaccine has been updated, on average, every 2.8 years (range 1 to 8 years), and the H3 component every 1.8 years (range 1 to 4 years) since 1980, it is reasonable to anticipate that A(H1N1)pdm09 viruses will undergo antigenic change in the near future.

At present oseltamivir remains the primary drug of choice for the treatment of human influenza infection worldwide, although the newly licensed neuraminidase inhibitor laninamivir has recently become widely used in Japan [58]. Until laninamivir becomes more widely available, oseltamivir will continue to remain the most accessible option for the prevention and treatment of influenza. Given the data presented here and recent reports of community-wide spread of oseltamivir resistant virus in the absence of drug selection pressure [23]–[25], there is an urgent need to reassess the almost exclusive reliance upon oseltamivir both for the treatment of human influenza infection and as the primary component of antiviral drug stockpiles for use during influenza pandemics. Alternatives include the other widely available influenza NA inhibitor drug, zanamivir, against which resistant viruses are rarely detected [59], and laninamivir, which is likely to become licensed and more widely available in coming years. It is notable that the majority of OR viruses (including those containing the NA H275Y mutation) retain sensitivity to zanamivir and laninamivir. Furthermore, future research efforts should investigate new antiviral drugs including those that target viral components other than the NA, which may be suitable for use alone or in combination with the current NA inhibitors [60].

Here we demonstrate that contemporary A(H1N1)pdm09 viruses have acquired NA mutations which permit the acquisition of NA H275Y without compromising viral fitness. These mutations, which are now present in virtually all circulating A(H1N1)pdm09 viruses, enhance the surface expression and enzymatic activity of the A(H1N1)pdm09 H275Y NA protein *in vitro* and result in enhanced viral fitness *in vivo*. Hence, the risk that H275Y A(H1N1)pdm09 viruses will spread globally, in a similar manner to OR seasonal A(H1N1) viruses in 2007–2008, now appears greater than at any time since the A(H1N1)pdm09 lineage emerged in 2009.

## Supporting Information

Figure S1 **Evolution of the NA V241I and N369K mutations in recent A(H1N1)pdm09 viruses.** A(H1N1)pdm09 protein sequences were downloaded from the Global Initiative on Sharing All Influenza Data website (http://www.gisaid.org) and the influenza virus resource at the National Centre for Biotechnology Information. The percentages of occurrences for each of the mutations using A/California/07/2009 as the reference strain were calculated on a monthly basis (based on the month of sample collection) since April 2009. Only mutations that were found in 100% of all circulating viruses in any of the months, as well as the H275Y and N386 mutations are shown.
(TIF)

Figure S2 **Oseltamivir susceptibility of HNE2011 A(H1N1)pdm09 viruses.** Confluent monolayers of MDCK cells were infected with either New17 OR or New163 OS virus at an MOI of 0.001. After 1 h incubation residual virus was removed and fresh medium ±250 nM oseltamivir was added to the monolayers. Supernatant samples were harvested 48 h pi and titrated on MDCK monolayers in 96-well plates. The presence or absence of haemagglutinating virus in each well was assessed four days later and virus titres calculated according to the method of Reed and Muench [29]. All results represent the mean titre ± the standard deviation for three replicates.
(TIF)

Figure S3 **QPCR analysis of New17 OR:New163 OS virus mixtures in ferrets.** Donor ferrets were infected with pure populations or virus mixtures of A/Newcastle/17/2011 oseltamivir resistant (New17 OR) and A/Newcastle/163/2011 oseltamivir sensitive (New163 OS). Daily nasal washes from donor and 1^st^ and 2^nd^ recipient ferrets were assayed to measure the viral replication and transmission kinetics of each virus mixture/pure population. The amount of viral RNA in each nasal wash was determined by real time RT-PCR quantification of the influenza matrix gene. Bars display the mean and standard deviation of three replicate RT-PCR assays.
(TIF)

Figure S4 **NA V241I and/or N369K addition/removal does not substantially affect viral replication of A(H1N1)pdm09 viruses** ***in vitro*** **.** Confluent MDCK monolayers were infected with viruses at a high and low multiplicity of infection (MOI) of 1 and 0.01 respectively. Thereafter virus infected supernatants were sampled at the time points indicated and titrated on MDCK monolayers in 96-well plates. The presence or absence of haemagglutinating virus in each well was assessed four days later and virus titres calculated according to the method of Reed and Muench [29]. (A) *in vitro* replication of New17 OR, rgNew17 OR and rgNew17 OR mutant viruses at 1 MOI. (B) *in vitro* replication of New 17 OR, rgNew17 OR and rgNew17 OR mutant viruses at 0.01 MOI. (C) *in vitro* replication of Perth261 OR, rgPerth261 OR and the rgPerth261 V241I, N369K OR double mutant virus at 1 MOI. (D) *in vitro* replication of Perth261 OR, rgPerth261 OR and the rgPerth261 V241I, N369K OR double mutant virus at 0.01 MOI. OR  =  Oseltamivir resistant.
(TIF)

Figure S5 **QPCR analysis of rgNew17 OR:rgNew17 I241V, K369N OR virus mixtures in ferrets.** Donor ferrets were infected with pure populations or virus mixtures of reverse genetics derived New17 OR (rgNew17 OR) and rgNew17 I241V, K369N OR. Daily nasal washes from donor, and naive 1st and 2nd recipient ferrets were assayed to measure the viral replication and transmission kinetics of each virus mixture/pure population. The amount of viral RNA in each nasal wash was determined by real time RT-PCR quantification of the influenza matrix gene. Bars display the mean and standard deviation of three replicate RT-PCR assays.
(TIF)

Figure S6 **Removal of NA V241I reduces the within-host and transmission fitness of recent H275Y A(H1N1)pdm09 viruses.** Donor ferrets were infected with pure populations or virus mixtures of rgNew17 OR and rgNew17 I241V OR. Daily nasal washes from donor and 1st and 2nd recipient ferrets were assayed to measure the viral replication and transmission kinetics of each virus mixture/pure population and to assess the relative proportions of each virus within mixtures. (A) The infectious virus titre in each nasal wash was determined by titration on MDCK cells. (B) The relative proportions of virus encoding NA 241I (black bars) and NA 241V (white bars) in each nasal wash were determined by pyrosequencing. (C) The amount of viral RNA in each nasal wash was determined by real time RT-PCR quantification of the influenza matrix gene. Bars display the mean and standard deviation of three replicate RT-PCR assays. (A, C) Virus in donor ferrets (grey), 1st recipient ferrets (black lines solid squares), 2nd recipient ferrets (black lines, white triangles).
(TIF)

Figure S7 **Removal of NA N369K substantially decreases the within-host fitness of recent H275Y A(H1N1)pdm09 viruses.** Donor ferrets were infected with pure populations or virus mixtures of rgNew17 OR and rgNew17 K369N OR. Daily nasal washes from donor and 1st and 2nd recipient ferrets were assayed to measure the viral replication and transmission kinetics of each virus mixture/pure population and to assess the relative proportions of each virus within mixtures. (A) The infectious virus titre in each nasal wash was determined by titration on MDCK cells. (B) The relative proportions of virus encoding NA 369K (black bars) and NA 369N (white bars) in each nasal wash were determined by pyrosequencing. (C) The amount of viral RNA in each nasal wash was determined by real time RT-PCR quantification of the influenza matrix gene. Bars display the mean and standard deviation of three replicate RT-PCR assays. (A, C) Virus in donor ferrets (grey), 1st recipient ferrets (black lines solid squares), 2nd recipient ferrets (black lines, white triangles).
(TIF)

Figure S8 **QPCR analysis of rgPerth261 OR:rgPerth261 V241I, N369K OR virus mixtures in ferrets.** Donor ferrets were infected with pure populations or virus mixtures of reverse genetics derived A/Perth/261/2009 oseltamivir resistant (rgPerth261 OR) and rgPerth261 V241I, N369K OR. Daily nasal washes from donor and 1st and 2nd recipient ferrets were assayed to measure the viral replication and transmission kinetics of each virus mixture/pure population. The amount of viral RNA in each nasal wash was determined by real time RT-PCR quantification of the influenza matrix gene. Bars display the mean and standard deviation of three replicate RT-PCR assays.
(TIF)

Table S1 **GISAID accession numbers for viruses used in this study.** a, OR  =  Oseltamivir resistant due to the NA H275Y mutation. b, OS  =  Oseltamivir sensitive.
(DOCX)

Table S2 **Primer sequences for RT-PCR and pyrosequencing.** a, F  =  Forward primer. b, Biotin  =  Primer was biotinylated at the 5′ end. c, R  =  Reverse primer. d, S  =  Sequencing primer for pyrosequencing.
(DOCX)

Table S3 **Oseltamivir sensitivity of viruses used in this study.** a, IC_50_  =  The concentration of oseltamivir required to inhibit virus growth by 50%. b, OR  =  Oseltamivir resistant due to the NA H275Y mutation. c, OS  =  Oseltamivir sensitive.
(DOCX)

Text S1 **Details of the mathematical model used to estimate the relative within-host replication and transmission fitness values for each virus pair.**
(PDF)

Text S2 **Acknowledgement information for the virus sequences downloaded from GISAID for database analysis of the potentially permissive mutations.**
(XLSX)
