# Supplementary figures and images for: Estimating the Fitness Advantage Conferred by Permissive Neuraminidase Mutations in Recent Oseltamivir-Resistant A(H1N1)pdm09 Influenza Viruses
Source: PLoS Pathog. 2014 Apr 3;10(4):e1004065. doi: 10.1371/journal.ppat.1004065 (PMC3974874; doi:10.1371/journal.ppat.1004065)

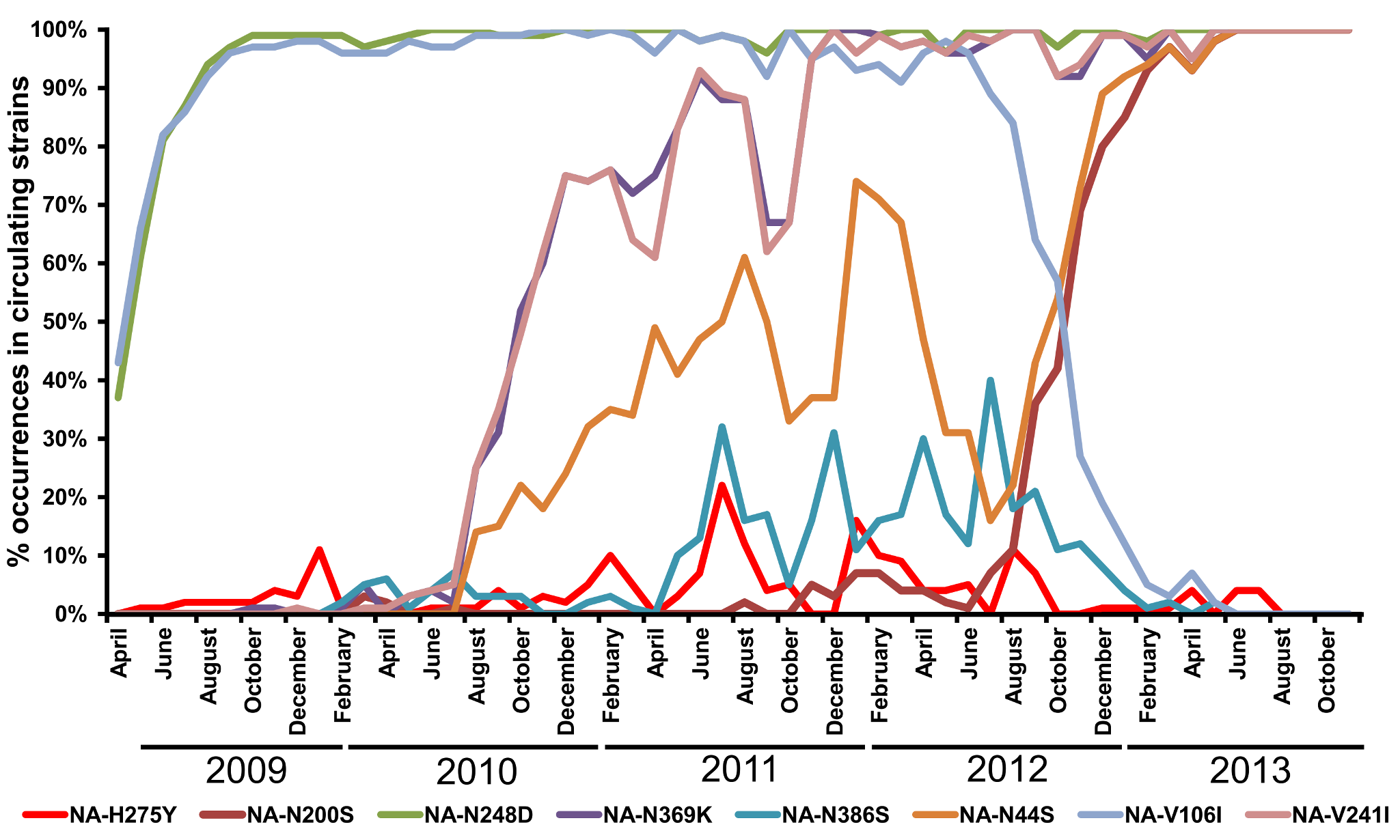

Supplement: Figure S1 — Evolution of the NA V241I and N369K mutations in recent A(H1N1)pdm09 viruses. A(H1N1)pdm09 protein sequences were downloaded from the Global Initiative on Sharing All Influenza Data website (http://www.gisaid.org) and the influenza virus resource at the National Centre for Biotechnology Information. The percentages of occurrences for each of the mutations using A/California/07/2009 as the reference strain were calculated on a monthly basis (based on the month of sample collection) since April 2009. Only mutations that were found in 100% of all circulating viruses in any of the months, as well as the H275Y and N386 mutations are shown. (TIF) [file ppat.1004065.s001.tif]

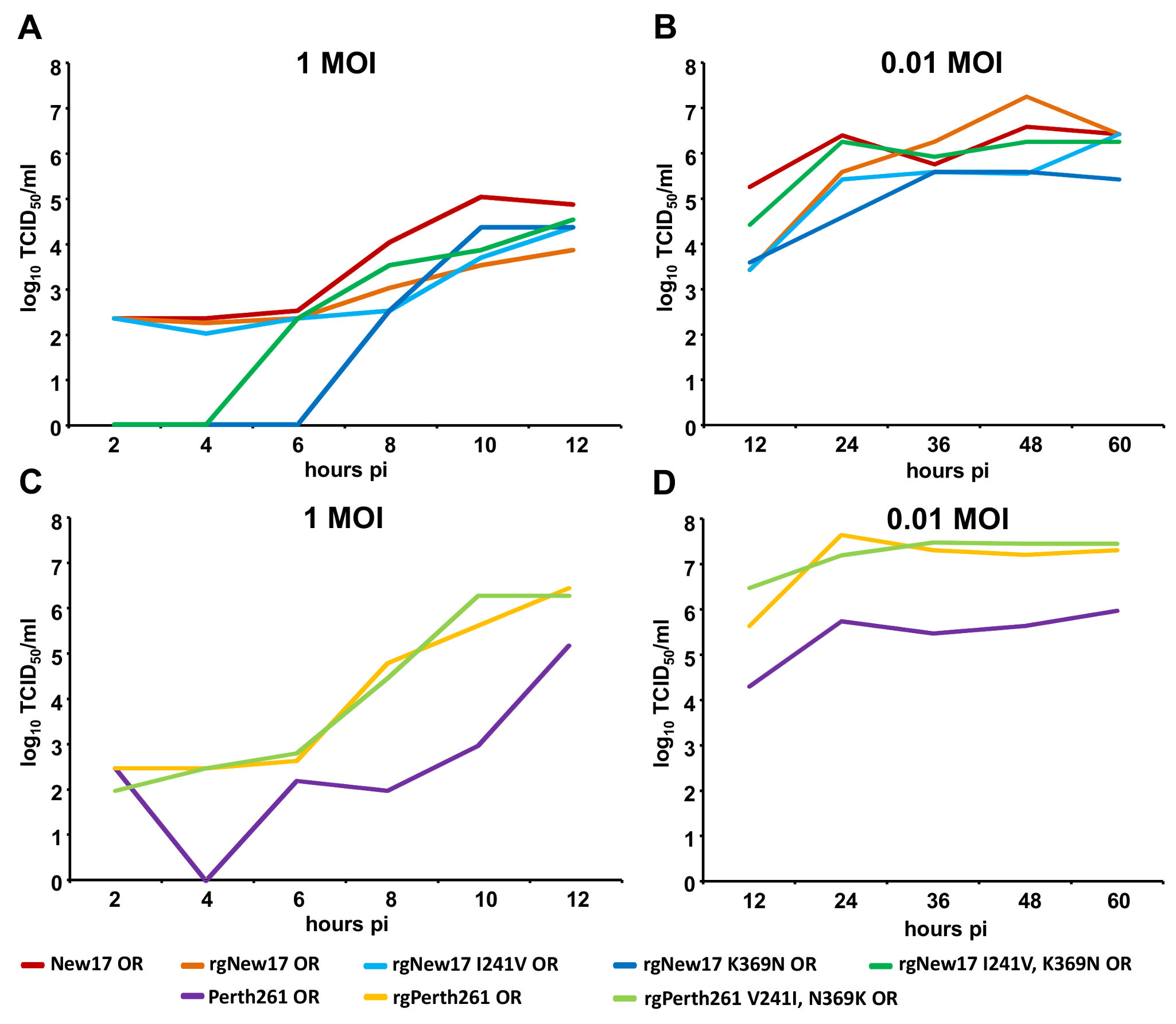

Supplement: Figure S4 — NA V241I and/or N369K addition/removal does not substantially affect viral replication of A(H1N1)pdm09 viruses in vitro . Confluent MDCK monolayers were infected with viruses at a high and low multiplicity of infection (MOI) of 1 and 0.01 respectively. Thereafter virus infected supernatants were sampled at the time points indicated and titrated on MDCK monolayers in 96-well plates. The presence or absence of haemagglutinating virus in each well was assessed four days later and virus titres calculated according to the method of Reed and Muench [29]. (A) in vitro replication of New17 OR, rgNew17 OR and rgNew17 OR mutant viruses at 1 MOI. (B) in vitro replication of New 17 OR, rgNew17 OR and rgNew17 OR mutant viruses at 0.01 MOI. (C) in vitro replication of Perth261 OR, rgPerth261 OR and the rgPerth261 V241I, N369K OR double mutant virus at 1 MOI. (D) in vitro replication of Perth261 OR, rgPerth261 OR and the rgPerth261 V241I, N369K OR double mutant virus at 0.01 MOI. OR = Oseltamivir resistant. (TIF) [file ppat.1004065.s004.tif]
