# Supplementary material for: Estimating the Fitness Advantage Conferred by Permissive Neuraminidase Mutations in Recent Oseltamivir-Resistant A(H1N1)pdm09 Influenza Viruses
Source: PLoS Pathog. 2014 Apr 3;10(4):e1004065. doi: 10.1371/journal.ppat.1004065 (PMC3974874; doi:10.1371/journal.ppat.1004065)
Supplement: Table S1 — GISAID accession numbers for viruses used in this study. a, OR = Oseltamivir resistant due to the NA H275Y mutation. b, OS = Oseltamivir sensitive. (DOCX) [file ppat.1004065.s009.docx]

| **Virus** | **Gene** | **GISAID accession number** |
| --- | --- | --- |
| New17 OR^a^ | PB2 | EPI465435 |
|  | PB2 | EPI465436 |
|  | PA | EPI465437 |
|  | HA | EPI334770 |
|  | NP | EPI465438 |
|  | NA | EPI334769 |
|  | M | EPI465439 |
|  | NS | EPI465440 |
| New163 OS^b^ | PB2 | EPI465441 |
|  | PB2 | EPI465442 |
|  | PA | EPI465443 |
|  | HA | EPI334788 |
|  | NP | EPI465444 |
|  | NA | EPI334787 |
|  | M | EPI465445 |
|  | NS | EPI465446 |
| Perth261 OR | PB2 | EPI269969 |
|  | PB2 | EPI346375 |
|  | PA | EPI346376 |
|  | HA | EPI269971 |
|  | NP | EPI346377 |
|  | NA | EPI269970 |
|  | M | EPI269968 |
|  | NS | EPI346378 |
