# Supplementary material for: Estimating the Fitness Advantage Conferred by Permissive Neuraminidase Mutations in Recent Oseltamivir-Resistant A(H1N1)pdm09 Influenza Viruses
Source: PLoS Pathog. 2014 Apr 3;10(4):e1004065. doi: 10.1371/journal.ppat.1004065 (PMC3974874; doi:10.1371/journal.ppat.1004065)
Supplement: Table S2 — Primer sequences for RT-PCR and pyrosequencing. a, F = Forward primer. b, Biotin = Primer was biotinylated at the 5′ end. c, R = Reverse primer. d, S = Sequencing primer for pyrosequencing. (DOCX) [file ppat.1004065.s010.docx]

| **Primer name** | **Sequence 5’ - 3’** | **Assay** | **Type of primer** |
| --- | --- | --- | --- |
| NA-H275Y-F^a^ | Biotin^b^-GACAGGCCTCATACAAGATCTTC | NA H275Y | RT‑PCR |
| NA-H275Y-R^c^ | ATCCCTGCACACACATGTGAT | NA H275Y | RT‑PCR |
| NA-H275Y-S^d^ | TAACAGGAGCATTCCTCATA | NA H275Y | Pyrosequencing |
| NA-V241I-F | Biotin-GAGAACACAAGAGTCTGAATGTG | NA V241I | RT‑PCR |
| NA-V241I-R | GATCTTGTATGAGGCCTGTCC | NA V241I | RT‑PCR |
| NA-V241I-S | CTTGGTCCATCGGTCATT | NA V241I | Pyrosequencing |
| NA-N369K-F | ACAGGCAGTTGTGGTCCAGTAT | NA N369K | RT‑PCR |
| NA-N369K-R | Biotin-TGGATCCCAAATCATCTCAAAAC | NA N369K | RT‑PCR |
| NA-N369K-S | CTAAAAGCATTAGTTCAAGA | NA N369K | Pyrosequencing |
