# Supplementary material for: Estimating the Fitness Advantage Conferred by Permissive Neuraminidase Mutations in Recent Oseltamivir-Resistant A(H1N1)pdm09 Influenza Viruses
Source: PLoS Pathog. 2014 Apr 3;10(4):e1004065. doi: 10.1371/journal.ppat.1004065 (PMC3974874; doi:10.1371/journal.ppat.1004065)
Supplement: Table S3 — Oseltamivir sensitivity of viruses used in this study. a, IC50 = The concentration of oseltamivir required to inhibit virus growth by 50%. b, OR = Oseltamivir resistant due to the NA H275Y mutation. c, OS = Oseltamivir sensitive. (DOCX) [file ppat.1004065.s011.docx]

| **Virus** | **NA 275 H/Y** | **IC_50_ (nM)^a^** | **Oseltamivir susceptibility** |
| --- | --- | --- | --- |
| New17 OR^b^ | Y | 223.0 ± 14.2 | Resistant |
| New163 OS^c^ | H | 3.7 ± 0.1 | Sensitive |
