# Supplementary material for: Estimating the Fitness Advantage Conferred by Permissive Neuraminidase Mutations in Recent Oseltamivir-Resistant A(H1N1)pdm09 Influenza Viruses
Source: PLoS Pathog. 2014 Apr 3;10(4):e1004065. doi: 10.1371/journal.ppat.1004065 (PMC3974874; doi:10.1371/journal.ppat.1004065)
Supplement: Text S1 — Details of the mathematical model used to estimate the relative within-host replication and transmission fitness values for each virus pair. (PDF) [file ppat.1004065.s012.pdf]

Supplementary Text S1

Mathematical model of competitive mixtures experiments

In order to model competitive mixtures experiments we extend the within-host model described by Petrie *et al.* [1] to account for co-infection by two competing viruses (A and B). Of note, the model is fit to both infectious and total (infectious and non-infectious) viral load, measured by the $TCID_{50}$ and RT-PCR assays respectively. The resulting model (Figure 1) is similar to an *in vitro* co-infection model developed by Pinilla *et al.* [2].

We assume that each virus (V_A^{TCID} and V_B^{TCID}) competes for the same target cell (T) resources:

$$\frac{dT}{dt} = -\beta_A T V_A^{TCID} - \beta_B T V_B^{TCID}. \tag{1}$$

Upon infection, target cells (T) progress through a latent phase (L) before becoming infectious (I) as described by the following equations:

$$\frac{dL_A}{dt} = \beta_A T V_A^{TCID} - k_A L_A \tag{2}$$

$$\frac{dL_B}{dt} = \beta_B T V_B^{TCID} - k_B L_B \tag{3}$$

$$\frac{dI_A}{dt} = k_A L_A - \delta_A I_A \tag{4}$$

$$\frac{dI_B}{dt} = k_B L_B - \delta_B I_B \tag{5}$$

$$\frac{dV_A^{TCID}}{dt} = p_A I_A - c_{h,A} V_A^{TCID} - d_{inf,A} V_A^{TCID} \tag{6}$$

$$\frac{dV_B^{TCID}}{dt} = p_B I_B - c_{h,B} V_B^{TCID} - d_{inf,B} V_B^{TCID} \tag{7}$$

$$\frac{dV_A^{RNA}}{dt} = \xi_A p_A I_A - c_{h,A} V_A^{RNA} \tag{8}$$

$$\frac{dV_B^{RNA}}{dt} = \xi_B p_B I_B - c_{h,B} V_B^{RNA}. \tag{9}$$

Details of all state variables and parameters in this model are shown in Table 1. The waiting time distribution in the L and I compartments is modelled by an Erlang distribution by splitting each state into 20 stages as previously described by Petrie *et al.* [1].

The experiments investigated mutations within the NA gene, and a primary function of NA is to release budded virus from the surface of infected cells [3, 4]. We therefore assumed that observed differences in within-host viral kinetics between strains arose through a difference in the production rate of infectious virus from infected cells ($p_A \neq p_B$). All other model parameters were assumed to be identical for both strains. The ratio of the production rates (p_B/p_A) serves as a measure of relative within-host fitness.

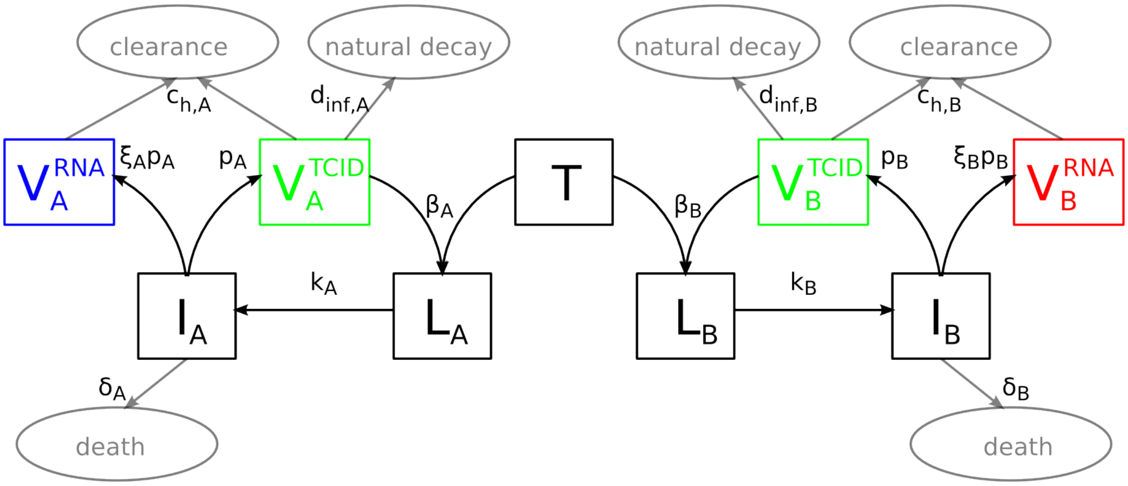

Figure 1: **Model schematic.** Schematic of the within-host co-infection model.

Fitting the model to experimental competitive-mixtures data

When fitting model outputs to experimental data, the infectious concentration of both viruses combined ($V_{comb}^{TCID} = V_A^{TCID} + V_B^{TCID}$) is fit to $TCID_{50}$ data, the viral RNA concentration of both strains combined ($V_{comb}^{RNA} = V_A^{RNA} + V_B^{RNA}$) is fit to RT-PCR data, and the proportion of viral RNA comprised of virus B (V_B^{RNA}/V_{comb}^{RNA}) is fit to pyrosequencing data.

We use identical initial conditions for T, L, I, and V_{comb}^{TCID} to those used previously by Petrie *et al.* [1], i.e.: $T(0) = 7 \times 10^7$, $L(0) = I(0) = 0$ (for each strain), and $V_{comb}^{TCID}(0)$ is a fitted parameter. For each mixture group we fix the initial infectious virus B proportion ($V_B^{TCID}(0)/V_{comb}^{TCID}(0)$) to the proportion prepared in the inoculum. The initial total:infectious proportion for both viruses combined ($V_{comb}^{RNA}(0)/V_{comb}^{TCID}(0)$) is fit to the RT-PCR:$TCID_{50}$ ratio as measured in the inoculum, and the initial total virus B proportion ($V_B^{RNA}(0)/V_{comb}^{RNA}(0)$) is fit to inoculum pyrosequencing data.

Model parameters are estimated by minimising the sum of squared residuals (SSR) between model outputs and data. MATLAB 2011b's genetic algorithm is used to ensure that the global minimum of the SSR is obtained and to generate non-parametric 95% confidence intervals for parameter estimates, as described previously by Petrie *et al.* [1].

Table 1: **Definitions of all state variables (compartments) and parameters in the model.**

	Description	Units
T	number of target cells	*cells*
L	number of latently infected cells	*cells*
I	number of productively infected cells	*cells*
V^{TCID}	concentration of free infectious virions measured via $TCID_{50}$ infectivity assay	$TCID_{50}/ml$ *of nasal wash*
V^{RNA}	concentration of total viral RNA (vRNA; present in both infectious and non-infectious virus) measured via RT-PCR assay	*vRNA copies/ml*
β	rate governing infection of target cells by infectious virions	$(TCID_{50}/ml)^{-1}\ d^{-1}$
p	rate of production of infectious virions	$(TCID_{50}/ml)\ cell^{-1}\ d^{-1}$
k	transition rate from latent to productive infection	d^{-1}
δ	death rate of productively infected cells	d^{-1}
c_h	host-driven clearance rate (assumed to be the same for both infectious and non-infectious viral particles)	d^{-1}
d_{inf}	rate of degradation of infectious virions to non-infectious viral particles (fixed to $d_{inf} = 3.12d^{-1}$; a value determined previously *in vitro* for two H1N1pdm09 viruses [2]).	d^{-1}
ξ	ratio of total vRNA measured via rRT-PCR to infectious virions measured via $TCID_{50}$, as produced by infected cells	*vRNA copies*$/TCID_{50}$

Alternative biological drivers of strain-dependence in viral kinetics

In using the within-host mathematical model to calculate the relative within-host viral replication fitness (Table 1, main results) we made the assumption that the observed strain-dependence in viral kinetics arose due to differing infectious virus production rates between strains. However there are other possibilities regarding strain-dependent biological processes that could generate differences in viral load kinetics and hence explain the experimental observations.

Several of these alternative explanations have been investigated recently *in vitro* for certain H275Y mutant strains and related wild-type strains [2], including the possibility that the duration of the latent period (the time between infection and release of viral progeny) differs between strains, or that the production rate of non-infectious virus differs between strains. However each of these hypotheses have limitations when investigating relative within-host fitness in the competitive mixtures experimental system.

The former is capable of generating scenarios in which co-infecting strains effectively do not compete for target cells (due to either large viral inocula infecting most available target cells prior to the production of any progeny virus within a host, or to each strain infecting very few target cells throughout the entire course of infection). The latter fundamentally assumes that there is no fitness difference between strains, as the within-host ratio of infectious virus by strain is assumed to be constant over time.

The potential for little or no competition between strains under either of these biological scenarios is inconsistent with the picture of outgrowth that we see across multiple host-to-host transmission events in many of the competitive-mixtures experiments performed in this work. A more complex model that describes within host viral kinetics and host-to-host transmission within a single framework, and which could be fitted simultaneously to data from both donor and recipient ferrets, may be required to appropriately explore such alternative biological hypotheses. Such a model is under active development.

References

[1] Petrie SM, Guarnaccia T, Laurie KL, Hurt AC, McVernon J, et al. (2013) Reducing uncertainty in within-host parameter estimates of influenza infection by measuring both infectious and total viral load. PloS ONE 8: e64098.

[2] Pinilla LT, Holder BP, Abed Y, Boivin G, Beauchemin CAA (2012) The H275Y Neuraminidase Mutation of the Pandemic A/H1N1 Influenza Virus Lengthens the Eclipse Phase and Reduces Viral Output of Infected Cells, Potentially Compromising Fitness in Ferrets. Journal of Virology 86: 10651–60.

[3] Colman PM (1994) Influenza virus neuraminidase: structure, antibodies, and inhibitors. Protein Science 3: 1687–96.

[4] Nayak DP, Hui EKW, Barman S (2004) Assembly and budding of influenza virus. Virus Research 106: 147–65.
